# Elucidation of the Role of Lectin-Like oxLDL Receptor-1 in the Metabolic Responses of Macrophages to Human oxLDL

**DOI:** 10.1155/2017/8479482

**Published:** 2017-05-31

**Authors:** Danielle W. Kimmel, William P. Dole, David E. Cliffel

**Affiliations:** ^1^Department of Chemistry, Vanderbilt University, VU Station B, Nashville, TN 37235-1822, USA; ^2^Vanderbilt Institute for Integrative Biosystems Research and Education, Vanderbilt University, Nashville, TN 37235-1809, USA; ^3^Novartis Institutes for Biomedical Research, 220 Massachusetts Ave. 360C, Cambridge, MA 02139, USA

## Abstract

Atherogenesis is the narrowing of arteries due to plaque build-up that results in cardiovascular disease that can lead to death. The macrophage lectin-like oxidized LDL receptor-1 (LOX-1), also called the oxidized low-density lipoprotein receptor 1 (OLR1), is currently thought to aid in atherosclerotic disease progression; therefore metabolic studies have potential to both provide mechanistic validation for the role of LOX-1 in disease progression and provide valuable information regarding biomarker strategies and clinical imaging. One such mechanistic study is the upregulation of LOX-1 by methylated bacterial DNA and deoxy-cytidylate-phosphate-deoxy-guanylate-DNA (CpG)-DNA exposure. CpG-DNA is known to promote oxidative burst responses in macrophages, due to its direct binding to toll-like receptor 9 (TLR9) leading to the initiation of an NF-*κ*B mediated immune response. In addition to the upregulation of macrophage LOX-1 expression, these studies have also examined the macrophage metabolic response to murine LOX-1/OLR1 antibody exposure. Our data suggests the antibody exposure effectively blocks LOX-1 dependent oxLDL metabolic activation of the macrophage, which was quantified using the multianalyte microphysiometer (MAMP). Using the MAMP to examine metabolic fluctuations during various types of oxLDL exposure, LOX-1 upregulation and inhibition provide valuable information regarding the role of LOX-1 in macrophage activation of oxidative burst.

## 1. Introduction

The uptake of oxidized low-density lipoprotein (oxLDL) is thought to promote atherogenesis and lead to clinically relevant cardiovascular diseases [1–4]. Our previous work using the MAMP showed that there is a correlation between oxLDL uptake by macrophages and increases in glucose and oxygen consumption and increases in lactate production and extracellular acidification rates [5]. Research suggests that macrophage uptake of oxLDL is regulated by cell surface atherogenic oxLDL receptors including class A macrophage scavenger receptors, class B type I scavenger receptors, macrosialin (CD68) [1, 3]. Previous research suggests the importance of the lectin-like oxidized low-density lipoprotein receptor-1 (LOX-1) [1, 3].

LOX-1 is a 50 kDa type II membrane glycoprotein composed of 273 amino acids [1, 3], which can selectively bind and internalize oxLDL [6]. Studies have shown that LOX-1 is expressed in macrophages among other cells but can also be stimulated in a variety of cell types using TNF-*α*, PMA, and shear stress [3]. The signaling pathway activated by LOX-1 promotes superoxide radical formation through the NADPH oxidase complex as well as promoting transcription of VCAM-1 through NF-*κ*B activation, both of which induce inflammatory responses leading to foam cell formation and atherogenesis [2, 4, 6]. Researchers have also studied LOX-1 deficient mice and subsequent knockout hybridization to find resistance toward oxLDL-induced arterial impairment, suggesting a vital role for LOX-1 in atherogenesis and arterial remodeling [6].

In the present study, our focus is to elucidate the role of LOX-1 in the biological response of murine macrophages to human oxLDL. Previous work has demonstrated that macrophages are a key cell type in atherosclerotic lesions [1, 3, 4, 6–8]. Their activity and responses to oxLDL particles play a major role in the pathogenesis of the disease. Understanding the metabolic responses of macrophages to oxLDL mediated by LOX-1 has the potential to both provide additional mechanistic validation of this receptor in disease processes and support clinical imaging and biomarker strategies for future investigation. Here, we utilize the MAMP to simultaneously measure glucose and oxygen consumption, lactate production, and extracellular acidification before, during, and after macrophage assault by oxLDL. This study focuses on the characterization of macrophage metabolic responses to various oxidation levels of LDL and the characterization of LOX-1 mediated effects of oxLDL on macrophage metabolism.

## 2. Materials and Methods

### 2.1. Materials

Unless otherwise noted, all materials used were previously reported in prior work [5]. In order to examine the differences in macrophage metabolic responses from various oxidation levels of LDL, an oxLDL kit was purchased by Novartis International (Boston, MA) from Kalen Biomedical (Montgomery Village, MD). The kit contained LDL, low oxLDL, medium oxLDL, and high oxLDL. Additional oxLDL was purchased through Intracel (Frederick, MD) for comparison with Kalen high oxLDL. Murine deoxy-cytidylate-phosphate-deoxy-guanylate- (CpG-) DNA was purchased through Hycult Biotech (Plymouth Meeting, PA). Mouse LOX-1/OLR1 antibody (MAB1564) and rat IgG2A isotype control (MAB006) were purchased through R&D Systems (Minneapolis, MN). Trehalose was purchased through Sigma-Aldrich (St. Louis, MO).

### 2.2. Methods

Cell culturing methods and experimental protocols were previously reported in prior work [5], all exceptions noted below. Initial oxLDL experiments were performed using each of the four types of LDL: native, low, medium, and highly oxidized. Throughout the course of multiple experiments, macrophages were exposed to each type of LDL at four concentrations: 1 *μ*g/mL, 10 *μ*g/mL, 50 *μ*g/mL, and 100 *μ*g/mL. Additionally, after an hour of stabilization time, macrophages were exposed to 25 *μ*g/mL mouse LOX-1/OLR1 antibody for 20 min, followed by 60 min of recovery to establish a baseline for the metabolic profile of the antibody. Macrophages were also exposed to 3 *μ*mol/L CpG-DNA for 30 min. For the studies examining the effects of mouse LOX-1/OLR1 antibody prior to oxLDL exposure, macrophages were exposed to 25 *μ*g/mL mouse LOX-1/OLR1 antibody for 10 min followed directly by a 6 min exposure to the 4 previously mentioned dosages of Kalen high oxLDL. Treatment and analysis of data were performed as previously reported [5].

## 3. Results

The present study is designed to investigate the relationship between the dynamic metabolic profiles of oxLDL uptake by macrophages with and without murine LOX-1/OLR1 antibodies present. To this end, it was vital to study the metabolic response of macrophages exposed to variations in oxidation of LDL as well as concentrations to ensure optimization of response before antibody exposure. To do this, experiments were executed using 6 min dosages of native LDL, low oxLDL, medium oxLDL, or high oxLDL at four concentrations: 1 *μ*g/mL, 10 *μ*g/mL, 50 *μ*g/mL, and 100 *μ*g/mL. During each experiment glucose and oxygen consumption, lactate production, and extracellular acidification were simultaneously measured to produce the dynamic response seen. Each data set was then compared with the baseline to assess significance. Additionally, a comparison of two commercially available oxLDL sources was performed to gauge which type incited a substantial and reproducible oxidative burst phenomenon. A *p* value of less than 0.05 was taken as statistically significant.

Native LDL was used as a baseline to determine if there were any significant variations in response during exposure or recovery due to the introduction of lipoproteins into the chamber. LDL exposure was performed at the four separate concentrations previously mentioned. Maximum changes in peak heights were taken during LDL exposure and compared with the baseline, prior to exposure. Using LDL response as a control, various forms of oxLDL were then studied and the resulting data was treated in the same manner. OxLDL from the Kalen Biomedical kit contained various forms of three different oxidation levels: low, medium, and high. In addition to the Kalen oxLDL, the Intracel version, with only one oxidation level, was also studied. For ease of comparison between LDL and each type of oxLDL each analyte is compiled into separate graphs.

**Figure 1:**
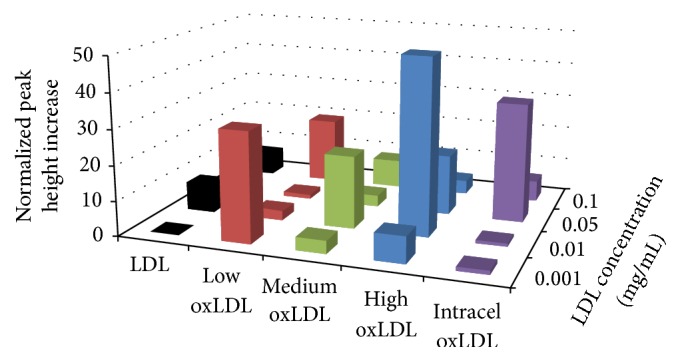
Average metabolic glucose consumption response of RAW 264.7 cells to a 6 min exposure of various LDL. Percent increases were calculated based on the variation from the maximum peak height chance during exposure to the baseline average, prior to exposure. For native LDL, the glucose consumption at 100 *μ*g/mL the number of averaged experiments (*n*) is 4 and the standard error of the mean for the basal metabolic rate of the baseline is ±0.20%. For 50 *μ*g/mL *n* = 2; ±0.27%, 10 *µ*g/mL *n* = 2; ±0.01%, and 1 *µ*g/mL *n* = 4; ±6.1%. For low oxLDL, 100 *μ*g/mL *n* = 3; ±12.5%, 50 *μ*g/mL *n* = 5; ±3.4%, 10 *µ*g/mL *n* = 3; ±0.36%, and 1 *µ*g/mL *n* = 2; ±0.38%. For medium oxLDL at 100 *μ*g/mL *n* = 2; ±0.33%, 50 *μ*g/mL *n* = 3; ±0.51%, 10 *µ*g/mL *n* = 4; ±0.16%, and 1 *µ*g/mL *n* = 5; ±0.32%. For high oxLDL at 100 *μ*g/mL *n* = 3; ±0.5%, 50 *µ*g/mL *n* = 3; ±9.7%, 10 *µ*g/mL *n* = 3; ±0.44%, and 1 *µ*g/mL *n* = 3; ±8.6%. For Intracel oxLDL at 100 *μ*g/mL *n* = 4; ±1.8%, 50 *µ*g/mL *n* = 6; ±15.4%, 10 *µ*g/mL *n* = 3; ±0.67%, and 1 *µ*g/mL *n* = 2; ±0.31%.

Glucose consumption by the macrophages during exposure showed great variation between native and oxidized LDL, as detailed in Figure 1 and Table 1. For native LDL, there were notable increases in glucose consumption for the 10 *μ*g/mL and 100 *μ*g/mL concentrations. Low oxLDL exposure did not reveal any significant increases in glucose consumption; however the medium oxLDL at 1 *μ*g/mL and 50 *μ*g/mL did show significance. While this does not show a clear trend in peak height increases for medium oxLDL exposures, it does showcase the increase in glucose consumption, which we have found to be a pivotal signature of oxLDL initiated oxidative burst. The moderately oxidized LDL is not providing the onset of foam cell formation but is inhibiting basal metabolism. High oxLDL and the Intracel oxLDL provided the clearest peak height increases, but with great biological variation causing an increase in standard error. The data suggests a rough trend of increasing glucose consumption based on an increase in oxidation of LDL. The decrease of glucose consumption with increasing concentrations of LDL exposure indicates that there might be receptor saturation preventing exponential update of glucose, thus limiting the onset of oxidative burst.

**Figure 2:**
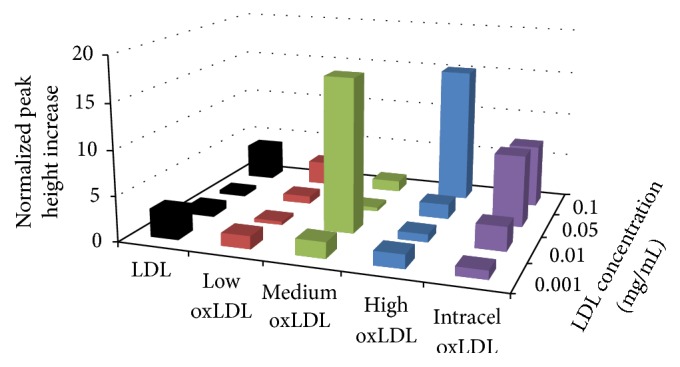
Average metabolic lactate production response of RAW 264.7 cells to a 6 min exposure of various LDL. Percent increases were calculated based on the variation from the maximum peak height chance during exposure to the baseline average, prior to exposure. For native LDL, the lactate production at 100 *µ*g/mL *n* = 3; ±0.01%, 50 *µ*g/mL *n* = 2; ±0.005%, 10 *µ*g/mL *n* = 5; ±0.35%, and 1 *µ*g/mL *n* = 4; ±0.04%. For low oxLDL at 100 *µ*g/ml *n* = 4; ±0.6%, 50 *µ*g/ml *n* = 4; ±0.12%, 10 *µ*g/ml  *n* = 5; ±0.14%, and 1 *µ*g/ml  *n* = 4; ±0.02%. For medium oxLDL at 100 *µ*g/ml *n* = 4; ±0.47%, 50 *µ*g/mL *n* = 4; ±4.6%, 10 *µ*g/mL *n* = 4; ±0.26%, and 1 *µ*g/mL *n* = 5; ±0.25%. For high oxLDL at 100 *µ*g/mL *n* = 3; ±0.67%, 50 *µ*g/mL *n* = 5; ±0.26%, 10 *µ*g/mL *n* = 3; ±0.12%, and 1 *µ*g/mL *n* = 4; ±0.9%. For Intracel oxLDL at 100 *µ*g/mL *n* = 5; ±0.03%, 50 *µ*g/mL *n* = 4; ±0.46%, 10 *µ*g/mL *n* = 4; ±0.51%, and 1 *µ*g/mL *n* = 4; ±0.55%.

A second analyte, lactate production, is also indicative of cellular metabolic health so it was simultaneously measured while macrophages underwent varying types of LDL exposures. The resulting data can be seen in Figure 2 and Table 2. Native LDL produced a statistically significant increase in response for the 1 *μ*g/mL and 100 *μ*g/mL concentration but exhibited no other trends toward oxidative burst. Using lowly oxidized LDL, a response was elicited for the 50 *μ*g/mL and 100 *μ*g/mL concentrations. This shows an increase in anaerobic metabolism, indicating that the macrophages have not significantly undergone oxidative burst during low oxLDL exposure, likely due to the lack of oxidation of the LDL. Both medium and highly oxidized LDL show limited responses, none of which are significant until the 100 *μ*g/ml concentration of medium oxLDL. This lack of lactate production increases shows that the cell is utilizing aerobic respiration normally, not necessarily indicative of undergoing oxidative burst. Finally, the Intracel oxLDL elicited responses at 10 *μ*g/mL, 50 *μ*g/mL, and 100 *μ*g/mL showing that it was effective at increasing aerobic respiration and thus promoting oxidative burst.

**Figure 3:**
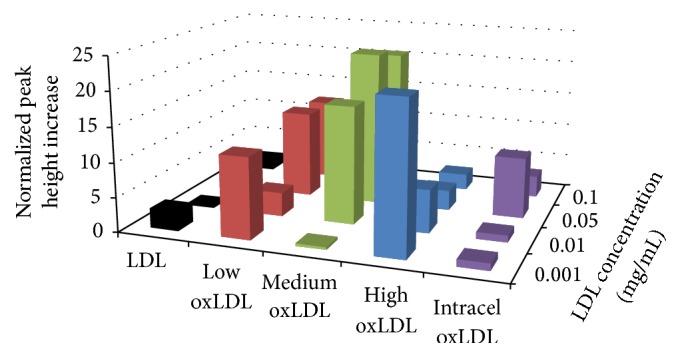
Average metabolic oxygen consumption response of RAW 264.7 cells to a 6 min exposure of various LDL. Percent increases were calculated based on the variation from the maximum peak height chance during exposure to the baseline average, prior to exposure. For native LDL at 100 *µ*g/mL *n* = 4; ±0.28%, 50 *µ*g/mL *n* = 3; ±0.27%, 10 *µ*g/mL *n* = 4; ±0.09%, and 1 *µ*g/mL *n* = 3; ±0.27%. For low oxLDL at 100 *µ*g/ml *n* = 2; ±1.03%, 50 *µ*g/ml *n* = 2; ±5.1%, 10 *µ*g/ml *n* = 3; ±0.56%, and 1 *µ*g/ml *n* = 3; ±0.33%. For medium oxLDL at 100 *µ*g/mL *n* = 3; ± 1.51%, 50 *µ*g/mL *n* = 4; ±0.74%, 10 *µ*g/mL *n* = 3; ±0.004%, and 1 *µ*g/mL *n* = 3; ±0.42%. For high oxLDL at 100 *µ*g/mL *n* = 2; ±0.37%, 50 *µ*g/mL *n* = 3; ±0.09%, 10 *µ*g/mL *n* = 3; ±0.11%, and 1 *µ*g/mL *n* = 2; ±0.94%. For Intracel oxLDL at 100 *µ*g/mL *n* = 4; ±0.16%, 50 *µ*g/mL *n* = 3; ±0.69%, 10 *µ*g/mL *n* = 3; ±0.57%, and 1 *µ*g/mL *n* = 4; ±0.28%.

During oxLDL exposure, increase in oxygen consumption coupled with increases in glucose consumption is indicative of oxidative burst onset. Data was collected from exposure to various forms of LDL and is presented in Figure 3 and Table 3. Native LDL exposure revealed statistically significant increases in oxygen consumption at 1 *µ*g/ml and 100 *µ*g/ml. The lack of an increasing trend of oxygen consumption during LDL exposure provides little evidence for oxidative burst onset. Kalen low and medium oxLDL exposure provided no statistically significant increases in oxygen consumption. However, both Kalen high oxLDL and the Intracel oxLDL provided a significant increase in oxygen consumption during the 50 *µ*g/ml. While there are limited statistically significant increases in oxygen consumption when compared with the baseline, biological samples promote wide variations in standard error. That said, there is an overall trend of increase in oxygen consumption with increase in oxidation level and concentration, up to a point. The higher levels of oxidation of LDL do not show this trend, suggesting a saturation of available receptors allowing for macrophage uptake.

**Figure 4:**
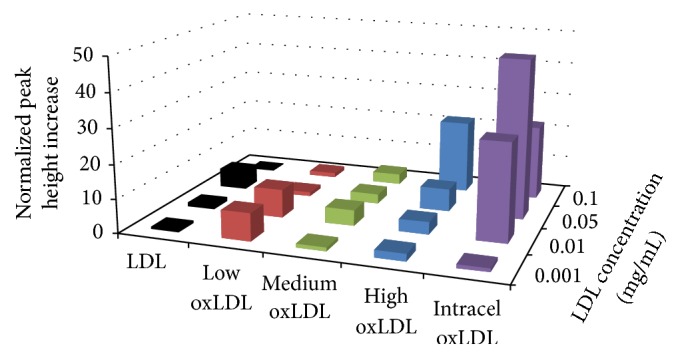
Average extracellular acidification response of RAW 264.7 cells to a 6 min exposure of various LDL. Percent increases were calculated based on the variation from the maximum peak height chance during exposure to the baseline average, prior to exposure. For native LDL at 100 *µ*g/mL *n* = 2; ±0.03%, 50 *µ*g/mL *n* = 3; ±0.15%, 10 *µ*g/mL *n* = 4; ±0.76%, and 1 *µ*g/mL *n* = 3; ±0.8%. For low oxLDL at 100 *µ*g/ml *n* = 3; ±0.9%, 50 *µ*g/ml *n* = 2; ±0.55%, 10 *µ*g/ml *n* = 5; ±8.8%, and 1 *µ*g/ml *n* = 2; ±0.24%. For medium oxLDL at 100 *µ*g/mL *n* = 2; ±0.22%, 50 *µ*g/mL *n* = 2; ±0.3%, 10 *µ*g/mL *n* = 2; ±0.003%, and 1 *µ*g/mL *n* = 3; ±0.78%. For high oxLDL at 100 *µ*g/mL *n* = 2; ±4.45%, 50 *µ*g/mL *n* = 3; ±4.3%, 10 *µ*g/mL *n* = 3; ±0.38%, and 1 *µ*g/mL *n* = 2; ±0.04%. For Intracel oxLDL at 100 *µ*g/mL *n* = 3; ±0.17%, 50 *µ*g/mL *n* = 3; ±1.0%, 10 *µ*g/mL *n* = 3; ±10.9%, and 1 *µ*g/mL *n* = 3; ±0.83%.

Extracellular acidification provides an overall view of the health of the cell during exposure to various forms of LDL. The data obtained during exposure is presented in Figure 4 and Table 4. Native LDL and low oxLDL provided statistically significant increases in extracellular acidification at the highest dosage concentration, 100 *µ*g/ml. The medium and high oxLDLs showed no dramatically significant increases in extracellular acidification; however the Intracel oxLDL showed an insignificant yet clear increasing trend with increasing dosages up to 50 *µ*g/ml. The extracellular acidification data does not provide conclusive evidence of oxidative burst onset; however extracellular acidification is the sum result of both anaerobic and aerobic metabolism. Therefore, this series does not adequately reflect the complexities of the macrophage metabolic response.

From the native LDL studies we see that LDL exposure induces a slight response seen primarily in glucose consumption and lactate production. This illustrates that the macrophage is increasing glucose uptake to ensure proper breakdown of the consumed LDL, which is highlighted by the increase in lactate production. These experiments allowed for a baseline metabolic response from the introduction of native LDL to compare with further experiments on macrophage exposure to various forms of oxLDL. The lowly oxidized LDL is not providing the onset of foam cell formation but is inhibiting basal metabolism. High oxLDL studies exhibited increases in metabolism signifying an increase in aerobic respiration, which is a hallmark of oxidative burst. This level of LDL oxidation showcases the ability of oxLDL to induce oxidative burst; however the lack of response for the highest dosage concentration also suggests an oversaturation of oxLDL receptors, inhibiting oxidative burst occurrence. There was also a marked increase seen with Intracel oxLDL, particularly at the 50 *µ*g/mL dosage level, indicating oxidative burst induction. The 100 *µ*g/mL dosage also has a response but is less intense than the 50 *µ*g/mL dosage, again suggesting oversaturation of oxLDL receptors.

**Figure 5:**
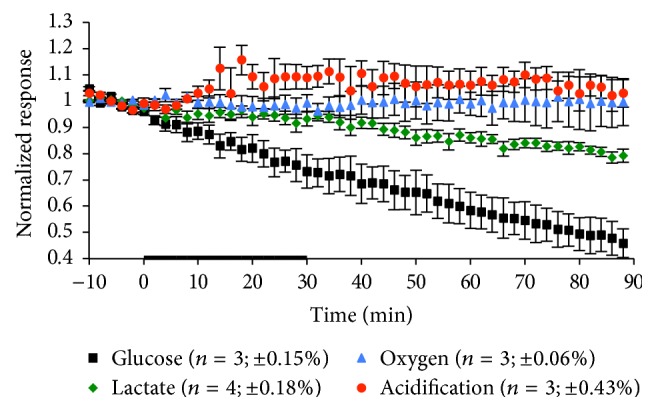
Average metabolic response of RAW 264.7 cells to a 30 min exposure of 3 *µ*mol/L CpG-DNA. The black bar indicates the exposure time. The number of experiments and standard error of the mean for the basal metabolic rate 10 min before exposure to CpG-DNA are given in parentheses.

Virulent components of pathogens can induce inflammatory responses that can be observed and measured by the MAMP. One known inducer and LOX-1 receptor upregulator is CpG-DNA, which directly binds to toll-like receptor 9 (TLR9) to promote an oxidative burst signaling cascade [9]. To incite a metabolic inflammatory response, macrophages were exposed to 3 *µ*mol/L CpG-DNA for 30 min (Figure 5, Table 5). During the exposure time, a significant peak height increase in glucose consumption and extracellular acidification was seen, indicating an increase in aerobic respiration to promote oxidative burst. During recovery significant peak height increases were also seen in lactate production. This suggests that after CpG-DNA is removed from the system, routine use of anaerobic and aerobic metabolism takes effect, thus leaving no lasting metabolic effects from the CpG-DNA exposure.

**Figure 6:**
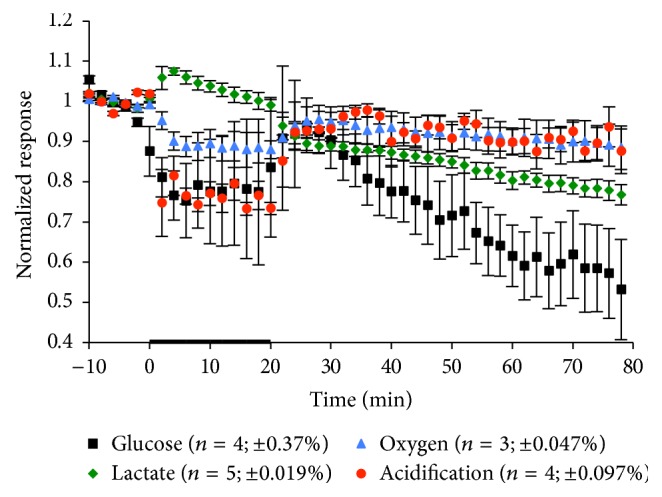
Average metabolic response of RAW 264.7 cells to a 20 min exposure of 25 *μ*g/mL mouse LOX-1/OLR1 antibody. The black bar indicates the exposure time. The number of experiments and standard error of the mean for the basal metabolic rate 10 min before exposure to the antibody are given in parentheses.

The murine LOX-1/OLR1 antibody is thought to completely block LOX-1 dependent oxLDL from binding to cells and will be utilized in concert with oxLDL exposure in later experiments. To ensure there are no lasting negative metabolic effects from the introduction of the antibody into the extracellular solution, we exposed macrophages to 25 *μ*g/mL of mouse LOX-1/OLR1 antibody for 20 min. The dynamic trace of the exposure and recovery is seen in Figure 6 and Table 6. Results indicated that, upon exposure, there were statistically significant decreases in glucose and oxygen consumption as well as extracellular acidification, while lactate production had a significant increase. The only significant decrease reported during recovery was with glucose consumption; however this decrease over time is often found in glucose consumption due to enzyme limitations of glucose oxidase. The rapid changes that occur during exposure indicate a decrease in aerobic respiration, suggesting an inhibition of oxidative burst. The inhibiting nature of the mouse LOX-1/OLR1 antibody toward oxidative burst may aid in oxidative burst prevention during oxLDL exposure.

**Figure 7:**
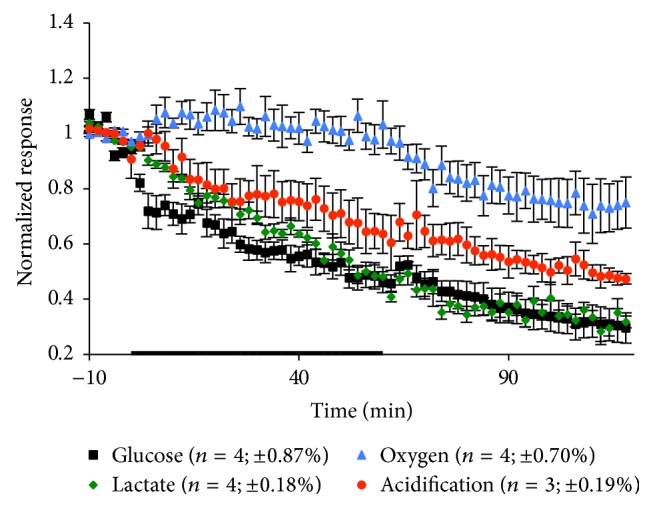
Average metabolic response of RAW 264.7 cells to a 60 min exposure of 50 mg/mL trehalose. The black bar indicates the exposure time. The number of experiments and standard error of the mean for the basal metabolic rate 10 min before exposure to the antibody are given in parentheses.

To ensure that the aforementioned change during mouse LOX-1/OLR1 antibody exposure was not due to trehalose, a concentration of 50 mg/mL was introduced to the cells for an hour to see if there was any significant effect. During the course of exposure, there were significant decreases seen in glucose consumption, lactate production, and extracellular acidification as can be seen in Figure 7 and Table 7. Compared with the changes seen during mouse LOX-1/OLR1 antibody exposure, there is not a recovery associated with trehalose exposure. Additionally, during trehalose exposure lactate production significantly decreases while oxygen consumption has an increasing trend, which is inverse during the mouse LOX-1/OLR1 antibody exposure. Therefore, this data suggests there is no lasting effect caused by trehalose presence within the mouse LOX-1/OLR1 antibody solution.

**Figure 8:**
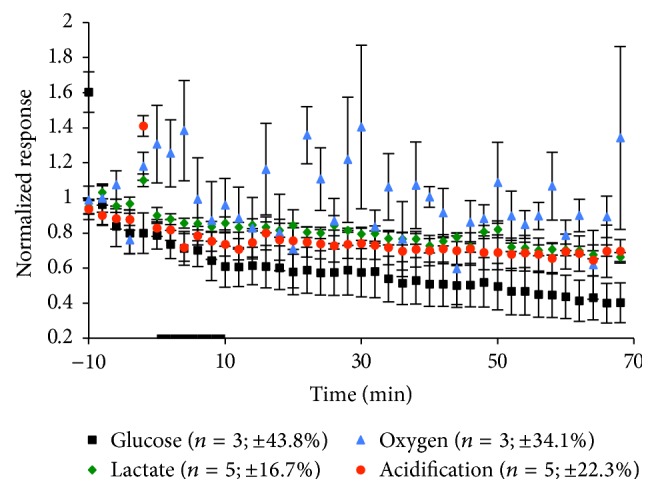
Average metabolic response of RAW 264.7 cells to a 10 min exposure of 25 *μ*g/mL rat IgG2A isotype control. The black bar indicates the exposure time. The number of experiments and standard error of the mean for the basal metabolic rate 10 min before exposure to the antibody are given in parentheses.

As a control, rat IgG2A isotype control was exposed to the cells at the same concentration as mouse LOX-1/OLR1 antibody, to ensure there were no significant changes in metabolism due to additive stability compounds. This data can be seen in Figure 8 and Table 8. There were no statistically significant metabolic changes during the 10 min exposure, suggesting the dramatic changes seen during mouse LOX-1/OLR1 antibody exposure are due solely to the antibody interaction with the macrophage.

Due to the ability of highly oxidized LDL to induce oxidative burst, Kalen high oxLDL was exposed to the macrophages after a 10 min exposure of mouse LOX-1/OLR1 antibody. This data can be seen in Table 9. Statistically significant lactate production changes from the basal metabolic rates that occurred during antibody exposure in the lower two dosages and oxygen consumption for the 100 *µ*g/mL dosage. These findings suggest that the antibody may not have significant deleterious effects on the macrophage metabolism when dosed in shorter time frames. Statistically significant changes from the basal rate were also seen during oxLDL exposure, but this was limited to two instances: lactate production at 10 *µ*g/mL and glucose consumption at 100 *µ*g/mL. The lack of statistically significant metabolic changes from the basal rate during antibody or oxLDL exposure, as well as during recovery, suggests that the antibody inhibited oxLDL to macrophage binding, thus prohibiting engulfment and oxidative burst.

## 4. Discussion

In the present study, we were able to identify peak height increases in metabolism during LDL exposure. Some anaerobic increases were statistically significant; however they were not seen in each analyte or to a high degree, indicating a lack of oxidative burst incitement. The metabolic hallmarks of oxidative burst are increases in all three metabolic analytes coupled with increases in the extracellular acidification. During LDL exposure, the most dramatic observable increase was in lactate production alone. This indicates that anaerobic respiration is upregulated while aerobic respiration remains fairly stable, which is not related to oxidative burst. Instead these changes indicate that lipoprotein exposure causes an increase in cellular uptake to promote LDL engulfment and subsequent breakdown for removal from the system.

In our previous study with oxLDL, significant responses were seen during or immediately following exposure. The oxLDL used in those studies was purchased through Intracel (Frederick, MD) who prepares each solution using human LDL. The differences in responses seen in this study could be caused from the variations in preparation techniques between Intracel and Kalen Biomedical, who report using varying oxidation conditions of human LDL for their oxLDL kit. However, there are responses, both statistically significant and nonsignificant, seen in the medium and high oxLDL types, suggesting an altering of the macrophage metabolism, likely due to the oxidative burst. Intracel oxLDL was also exposed to the macrophages at all four dosages and compared with the high oxLDL from Kalen. The responses were similar and indicated oxidative burst occurrence. Most notably, in both species of oxLDL the lower dosages exhibited greater changes in metabolism than the highest dosage. This data suggests the macrophage oxLDL receptors are saturated when exposed to higher concentrations of oxLDL, prohibiting oxidative burst from occurring.

The slight significant responses seen during CpG-DNA exposure do not follow expected oxidative burst metabolism. Current literature suggests that there is an acute inflammatory response to CpG-DNA due to its ability to overexpress LOX-1 receptors, but unfortunately reported exposure times vary [9]. Further experiments should examine longer exposure times and basal metabolic rates of macrophages that have been preincubated with CpG-DNA. Alternate concentrations of CpG-DNA should also be used to ensure maximal inflammatory response is gained.

Significant and rapid responses were seen upon mouse LOX-1/OLR1 antibody exposure. The responses seen indicate a rapid decrease in aerobic respiration, inhibiting oxidative burst. Control studies suggested that the response seen during mouse LOX-1/OLR1 antibody exposure is due to the antibody and not due to stability additives. This immediate response is likely due to the mouse LOX-1/OLR1 antibody interaction with the LOX-1 receptor, which typically interacts with oxLDL to promote cellular uptake and oxidative burst. Interestingly, the inhibition of aerobic respiration shows that the antibody/LOX-1 receptor interaction is actively altering cellular metabolism, suggesting LOX-1 receptor availability is vital to basal macrophage respiration. Further studies should be undertaken to assess the importance of LOX-1 to basal metabolism. Other studies should investigate various dosage concentrations of mouse LOX-1/OLR1 antibody to avoid oversaturation of the receptors.

Finally, preexposure of the macrophages with the mouse LOX-1/OLR1 antibody for a shorter time frame (10 min) showed limited metabolic responses during the mouse LOX-1/OLR1 antibody exposure. A subsequent high oxLDL dosage also showed limited changes of metabolic responses from the baseline indicating the mouse LOX-1/OLR1 antibody successfully prohibited significantly high oxLDL uptake that would lead to oxidative burst and foam cell formation.

## Figures and Tables

**Table 1:** Metabolic glucose consumption of various LDL exposure to macrophages. Mean peak MAMP height changes and standard errors for each experimental condition during exposure. All *p* values are calculated based on peak height versus 10 min average basal value prior to exposure. Terms in bold are representative of *p* < 0.05, indicating statistical significance.

Glucose					
	Native LDL (%)	Low oxLDL (%)	Medium oxLDL (%)	High oxLDL (%)	Intracel oxLDL (%)
1 *µ*g/ml	0.14 ± 18.3	31.2 ± 32.3	**3.67 ± 1.4**	7.5 ± 4.4	7.63 ± 12.4
	*p* = 0.9944	*p* = 0.3793	**p = 0.0348**	*p* = 0.5992	*p* = 0.582
10 *µ*g/ml	**8.2 ± 2.0**	2.5 ± 3.9	20.8 ± 22.0	56.4 ± 59.3	0.8 ± 3.2
	**p = 0.0263**	*p* = 0.5498	*p* = 0.377	*p* = 0.3792	*p* = 0.8164
50 *µ*g/ml	5.9 ± 10.0	1.3 ± 5.5	**3.19 ± 0.41**	30.6 ± 22.3	34.12 ± 29.8
	*p* = 0.5968	*p* = 0.8451	**p = 0.0046**	*p* = 0.2964	*p* = 0.3309
100 *µ*g/ml	**6.6 ± 1.4**	19.1 ± 17.8	8.3 ± 2.6	3.93 ± 4.07	**5.91 ± 1.5**
	**p = 0.0023**	*p* = 0.4201	*p* = 0.0506	*p* = 0.3785	**p = 0.0397**

**Table 2:** Metabolic lactate production of various LDL exposure to macrophages. Mean peak MAMP height changes and standard errors for each experimental condition during exposure. All *p* values are calculated based on peak height versus 10 min average basal value prior to exposure. Terms in bold are representative of *p* < 0.05, indicating statistical significance.

Lactate					
	Native LDL (%)	Low oxLDL (%)	Medium oxLDL (%)	High oxLDL (%)	Intracel oxLDL (%)
1 *µ*g/ml	**3.2 ± 0.68**	1.42 ± 1.58	1.81 ± 1.3	1.54 ± 2.8	2.0 ± 2.1
	**p = 0.0022**	*p* = 0.3987	*p* = 0.2047	*p* = 0.6001	*p* = 0.3875
10 *µ*g/ml	1.0 ± 1.0	0.39 ± 1.5	17.2 ± 20.0	0.91 ± 0.63	**2.7 ± 0.66**
	*p* = 0.3699	*p* = 0.8039	*p* = 0.4134	*p* = 0.2198	**p = 0.0143**
50 *µ*g/ml	0.47 ± 1.1	**0.84 ± 0.16**	0.38 ± 3.8	2.0 ± 1.8	**8.1 ± 2.35**
	*p* = 0.698	**p = 0.0040**	*p* = 0.9534	*p* = 0.3012	**p = 0.0117**
100 *µ*g/ml	**4.0 ± 1.5**	**2.7 ± 1.1**	1.21 ± 1.2	**15.2 ± 4.29**	**7.03 ± 3.0**
	**p = 0.0445**	**p = 0.0441**	*p* = 0.379	**p = 0.0172**	**p = 0.0438**

**Table 3:** Metabolic oxygen consumption of various LDL exposure to macrophages. Mean peak MAMP height changes and standard errors for each experimental condition during exposure. All *p* values are calculated based on peak height versus 10 min average basal value prior to exposure. Terms in bold are representative of *p* < 0.05, indicating statistical significance.

Oxygen					
	Native LDL (%)	Low oxLDL (%)	Medium oxLDL (%)	High oxLDL (%)	Intracel oxLDL (%)
1 *µ*g/ml	**2.9 ± 0.36**	11.8 ± 6.3	0.39 ± 0.72	21.7 ± 20.3	1.6 ± 4.2
	**p = 0.0013**	*p* = 0.1053	*p* = 0.6596	*p* = 0.3639	*p* = 0.7151
10 *µ*g/ml	0.3 ± 1.2	3.47 ± 5.9	17.1 ± 14.1	6.27 ± 6.55	1.0 ± 1.65
	*p* = 0.8103	*p* = 0.8338	*p* = 0.2794	*p* = 0.3822	*p* = 0.5915
50 *µ*g/ml	1.3 ± 2.2	12.8 ± 11.2	22.6 ± 20.7	**2.8 ± 0.78**	**8.88 ± 1.47**
	*p* = 0.583	*p* = 0.3748	*p* = 0.3114	**p = 0.0161**	**p = 0.0028**
100 *µ*g/ml	**1.7 ± 0.6**	12.1 ± 15.9	20.7 ± 18.2	2.36 ± 1.21	3.14 ± 1.7
	**p = 0.0371**	*p* = 0.5028	*p* = 0.3084	*p* = 0.1594	*p* = 0.1085

**Table 4:** Extracellular acidification rates of various LDL exposure to macrophages. Mean peak MAMP height changes and standard errors for each experimental condition during exposure. All *p* values are calculated based on peak height versus 10 min average basal value prior to exposure. Terms in bold are representative of *p* < 0.05, indicating statistical significance.

Acidification					
	Native LDL (%)	Low oxLDL (%)	Medium oxLDL (%)	High oxLDL (%)	Intracel oxLDL (%)
1 *µ*g/ml	0.73 ± 1.6	8.1 ± 3.3	1.03 ± 4.7	2.13 ± 1.7	2.13 ± 1.8
	*p* = 0.7001	*p* = 0.0918	*p* = 0.8452	*p* = 0.268	*p* = 0.3317
10 *µ*g/ml	1.2 ± 2.4	8.28 ± 6.6	4.53 ± 8.4	3.61 ± 4.8	28.5 ± 22.4
	*p* = 0.6481	*p* = 0.4708	*p* = 0.6254	*p* = 0.4864	*p* = 0.3044
50 *µ*g/ml	5.8 ± 5.2	1.12 ± 1.96	**2.8 ± 0.60**	13.1 ± 11.3	46.8 ± 23.6
	*p* = 0.3156	*p* = 0.6205	**p = 0.025**	*p* = 0.3280	*p* = 0.1044
100 *µ*g/ml	**25.6 ± 2.4**	**1.3 ± 0.1**	3.1 ± 5.2	21.6 ± 10.6	**22.0 ± 2.7**
	**p = 0.0018**	**p < 0.0001**	*p* = 0.8183	*p* = 0.2628	**p = 0.0005**

**Table 5:** Metabolic effects of CpG-DNA exposure on macrophages. Mean peak MAMP height changes and standard errors for each experimental condition during exposure and recovery are shown. All *p* values are calculated based on peak height versus 10 min average basal value prior to exposure. Terms in bold are representative of *p* < 0.05, indicating statistical significance.

CpG-DNA				
	Glucose (%)	Lactate (%)	Oxygen (%)	Acidification (%)
During Exposure	**4.0 ± 1.0**	1.4 ± 1.0	2.3 ± 2.5	**15.6 ± 5.6**
	**p = 0.0108**	*p* = 0.2107	*p* = 0.3999	**p = 0.039**
Recovery	**27.0 ± 5.3**	**5.6 ± 1.8**	1.6 ± 9.5	11.3 ± 4.8
	**p = 0.0038**	**p = 0.0174**	*p* = 0.8729	*p* = 0.066

**Table 6:** Metabolic effects of mouse LOX-1/OLR1 antibody exposure on macrophages. Mean peak MAMP height changes and standard errors for each experimental condition during exposure and recovery are shown. All *p* values are calculated based on peak height versus 10 min average basal value prior to exposure. Terms in bold are representative of *p* < 0.05, indicating statistical significance.

Mouse LOX-1/OLR1 antibody				
	Glucose (%)	Lactate (%)	Oxygen (%)	Acidification (%)
During exposure	−24.7 ± −9.2	7.4 ± 2.3	−12.1 ± −4.4	−26.7 ± −1.6
	**p = 0.0314**	**p = 0.0105**	**p = 0.0105**	**p = 6.9E** ^−**7**^
Recovery	−46.8 ± −12.5	6.2 ± 3.5	−11.6 ± −5.4	−14.8 ± −6.5
	**p = 0.0072**	*p* = 0.1103	*p* = 0.0845	*p* = 0.0569

**Table 7:** Metabolic effects of trehalose exposure on macrophages. Mean peak MAMP height changes and standard errors for each experimental condition during exposure and recovery are shown. All *p* values are calculated based on peak height versus 10 min average basal value prior to exposure. Terms in bold are representative of *p* < 0.05, indicating statistical significance.

Trehalose exposure				
	Glucose (%)	Lactate (%)	Oxygen (%)	Acidification (%)
During exposure	**53.9 ± 3.79**	**51.9 ± 3.53**	9.72 **±** 6.48	**36.4 ± 7.08**
	**p < 0.0001**	**p < 0.0001 **	*p* = 0.1815	**p = 0.0037**

**Table 8:** Metabolic effects of 25 *μ*g/mL Rat IgG2A Isotype Control exposure on macrophages. Mean peak MAMP height changes and standard errors for each experimental condition during exposure and recovery are shown. All *p* values are calculated based on peak height versus 10 min average basal value prior to exposure.

Rat IgG2A isotype control exposure				
	Glucose (%)	Lactate (%)	Oxygen (%)	Acidification (%)
During exposure	39.2 ± 11.7	16.6 ± 3.1	38.4 ± 28.7	28.2 ± 7.9
	*p* = 0.4268	*p* = 0.3539	*p* = 0.4283	*p* = 0.2637

**Table 9:** Metabolic effects of mouse LOX-1/OLR1 antibody 10 min exposure followed by 6 min exposure to Kalen high oxLDL on macrophages. Mean peak MAMP height changes and standard errors for each experimental condition during antibody exposure, oxLDL exposure, and recovery are shown. All *p* values are calculated based on peak height versus 10 min average basal value prior to exposure. Terms in bold are representative of *p* < 0.05, indicating statistical significance.

Mouse LOX-1/OLR1 antibody and oxLDL exposure					
		Glucose (%)	Lactate (%)	Oxygen (%)	Acidification (%)
1 *μ*g/mL		*n* = 3	*n* = 3	*n* = 3	*n* = 4
	Antibody exposure	20.1 ± 17.8	**3.15 ± 0.6**	43.2 ± 39.9	7.9 ± 7.7
		*p* = 0.9699	**p = 0.0046**	*p* = 0.3284	*p* = 0.4077
	oxLDL exposure	32.9 ± 34.3	5.1 ± 2.1	20.4 ± 13.3	5.27 ± 6.5
		*p* = 0.446	*p* = 0.0606	*p* = 0.1868	*p* = 0.5293
	Recovery	27.3 ± 44.0	**6.61 ± 1.9**	92.7 ± 56.1	29.7 ± 34.5
		*p* = 0.5972	**p = 0.0192**	*p* = 0.1594	*p* = 0.4217

10 *μ*g/mL		*n* = 3	*n* = 4	*n* = 4	*n* = 2
	Antibody exposure	1.92 ± 11.5	**3.55 ± 0.91**	17.34 ± 9.8	1.72 ± 0.97
		*p* = 0.9584	**p = 0.0109**	*p* = 0.1785	*p* = 0.1754
	oxLDL exposure	14.8 ± 27.1	**6.4 ± 0.99**	10.8 ± 2.6	11.2 ± 4.2
		*p* = 0.7436	**p = 0.0007**	*p* = 0.1529	*p* = 0.0759
	Recovery	12.3 ± 38.1	**9.9 ± 1.1**	0.2 ± 1.4	9.64 ± 6.9
		*p* = 0.8174	**p < 0.0001**	*p* = 0.9758	*p* = 0.2568

50 *μ*g/mL		*n* = 4	*n* = 3	*n* = 2	*n* = 2
	Antibody exposure	15.5 ± 20.8	5.7 ± 9.5	124.21 ± 124.2	4.7 ± 1.9
		*p* = 0.781	*p* = 0.5755	*p* = 0.391	*p* = 0.0995
	oxLDL exposure	14.0 ± 15.9	11.8 ± 8.9	141.9 ± 173.6	20.6 ± 10.9
		*p* = 0.7947	*p* = 0.2425	*p* = 0.4737	*p* = 0.1557
	Recovery	138.7 ± 104.2	1.1 ± 5.6	135.8 ± 166.9	**40.2 ± 6.2**
		*p* = 0.2679	*p* = 0.8496	*p* = 0.4754	**p = 0.0076**

100 *μ*g/mL		*n* = 3	*n* = 3	*n* = 3	*n* = 2
	Antibody exposure	1.85 ± 6.7	0.67 ± 0.25	**34.4 ± 10.2**	1.05 ± 1.51
		*p* = 0.7939	*p* = 0.2218	**p = 0.0203**	*p* = 0.5369
	oxLDL exposure	**8.56 ± 1.8**	4.34 ± 2.4	30.4 ± 12.1	34.6 ± 30.1
		**p = 0.0057**	*p* = 0.1348	*p* = 0.0544	*p* = 0.3337
	Recovery	**18.5 ± 1.9**	2.06 ± 1.9	10.3 ± 8.1	57.2 ± 48.6
		**p = 0.0002**	*p* = 0.3377	*p* = 0.2633	*p* = 0.324
